# Physiological evaluations of low-level impulsive sounds generated by an air conditioner

**DOI:** 10.3389/fpsyg.2023.1128752

**Published:** 2023-02-10

**Authors:** Yoshiharu Soeta, Ei Onogawa

**Affiliations:** ^1^Biomedical Research Institute, National Institute of Advanced Industrial Science and Technology (AIST), Osaka, Japan; ^2^Research and Innovation Center, Mitsubishi Heavy Industries Ltd., Nagoya, Aichi, Japan

**Keywords:** air conditioner, auditory evoked response, autocorrelation function, impulsive sound, sound quality

## Abstract

Air conditioners are typically installed in buildings and vehicles to control thermal conditions for long periods of time. Air conditioners generate certain types of sounds while functioning, which are among the main noise sources in buildings and vehicles. Most sounds produced by the air conditioner do not change with time, and the sound quality of steady sounds has been investigated. However, air conditioners can generate low-level impulsive sounds. Customers complain of the discomfort caused when these sounds disturb the silence in their living rooms and bedrooms. This study aimed to determine the physical factors that have a significant effect on physiological responses to low-level impulsive sounds produced by air conditioners. We used physiological responses because it is difficult for people to evaluate sounds psychologically when they are sleeping or are not focused on the sounds. The A-weighted equivalent continuous sound pressure level (L_Aeq_) and the factors extracted from the autocorrelation function (ACF) were evaluated as physical factors. Participant responses on electroencephalography (EEG) were evaluated. The correlation between the EEG responses and ACF factors was determined. The L_Aeq_, peak, and delay time to the first maximum peak of the ACF were identified as significant factors for physiological responses to low-level impulsive sounds.

## 1. Introduction

Air conditioners are used to maintain thermal comfort in almost all indoor environments. In the absence of a severe malfunction, the device generates a sound that is not salient. Although previous studies have indicated that the sounds generated by air conditioners can cause discomfort in laboratory environments (Blazier, 1997; Ayr et al., 2001; Tang and Wong, 2004; Forouharmajd et al., 2012; Nitschke et al., 2014; Soeta and Shimokura, 2017; Love et al., 2021; Soeta and Onogawa, 2021), most people do not mind the sound in real-life environments because of competing noise sources. However, air conditioners occasionally generate low-level impulsive sounds, which cause users to complain when they impact quiet living rooms and bedrooms (Soeta et al., 2023). Therefore, to increase user satisfaction, low-level impulsive sounds from air conditioners require evaluation.

The effects of low-level impulsive sounds from air conditioners on psychological responses of users have rarely been investigated. Previous studies on this topic have focused on tonal components, that is, noticeable narrowband components, because narrowband components are potentially more annoying than broadband components of the same sound pressure level (SPL). A previous study (Oliva et al., 2017; Yonemura et al., 2021) used various levels and frequencies of tonal components and proposed a level correction, that is, a penalty or tonal adjustment, for annoyance caused by the tonal component. A mathematical model for predicting such a correction was proposed (Hongisto et al., 2019). Previous studies assumed that tonal components were stable and used pure tones with durations of 9 or 10 s.

Exposure to high-level impulsive sounds may cause serious damage to hearing ability. The effects of peak level and duration have been investigated extensively (Henderson and Hamernik, 1986; Patterson, 1991; Chan et al., 2001). Effects of peak level, duration, and decay time on the loudness of impulsive sounds have also been investigated (Kuwano et al., 1987; Namba et al., 1987). Psychological responses to floor impact sounds were studied worldwide because they are typical impulsive sounds in indoor environments (Jeon et al., 2006; Frescura et al., 2022). Impulsive sounds, such as door closing and push-button sounds, have been attracting attention from the viewpoint of product sound quality. Acoustic characteristics and the subjective responses to sounds have been investigated (Kuwano et al., 2006; Parizet et al., 2008; Bezat et al., 2014; Gaspar et al., 2016; Valverde et al., 2018; Altinsoy, 2020). However, low-level impulsive sounds have rarely been addressed in previous studies.

From a physiological perspective, few studies have evaluated brain responses to impulsive sounds. Some studies have investigated brain responses to short sounds, such as click trains and tone bursts (Onishi and Davis, 1968; Kodera et al., 1979; Reite et al., 1982; Gage and Roberts, 2000; Lütkenhöner, 2011; Altinsoy, 2020). Previous studies have mainly focused on temporal integration and thresholds in relation to duration and level. The effects of changes in sound level (Martin and Boothroyd, 2000; Dimitrijevic et al., 2009; Nishihara et al., 2011; He et al., 2012; Soeta and Nakagawa, 2012), frequency (Martin and Boothroyd, 2000; Dimitrijevic et al., 2008; Pratt et al., 2009; Yamashiro et al., 2011; He et al., 2012; Weise et al., 2012, 2018; Vonck et al., 2019; Zhang et al., 2021), and location (Zhang et al., 2021; Fan et al., 2022) on brain responses have been investigated. A larger change caused larger N1 and P2 responses, which are typical responses of auditory evoked potentials (AEPs) and fields. Simple stimuli such as pure tone, harmonics, and synthesized vowels have been used. Previous studies only evaluated changes in only one or two components, and the sound was stable before and after the change. One previous psychological study showed the effects of changes in some sound components produced by air conditioners (Soeta et al., 2023). Such real sounds have rarely been used in physiological studies.

The present study aimed to identify indicators that correlate with physiological responses to low-level impulsive sounds. Psychological experiments require participants to pay attention to sounds, but in real-life, humans tend not to pay attention to all sounds. Thus, we used a physiological response, AEPs, to evaluate sounds in a more realistic situation. Autocorrelation function (ACF) factors were utilized, because our previous study indicated that the ACF factors were significantly correlated with psychological responses to low-level impulsive sounds (Soeta et al., 2023).

## 2. Methods

### 2.1. Measurement and analysis of air conditioner sounds

**Figure 1:**
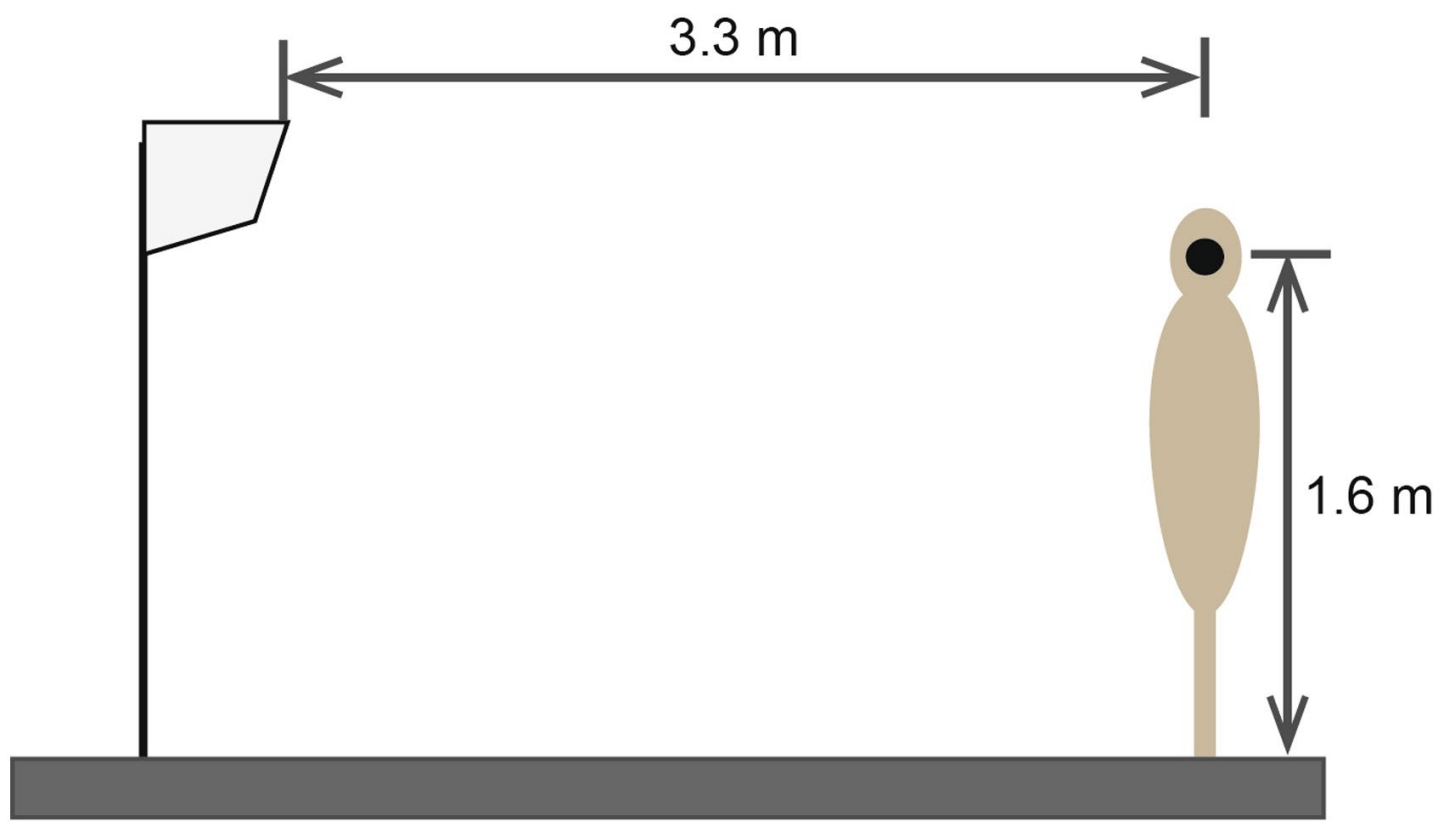
Installation of air conditioner units in an anechoic room. The binaural microphone was placed at the ear position on the mannequin.

**Figure 2:**
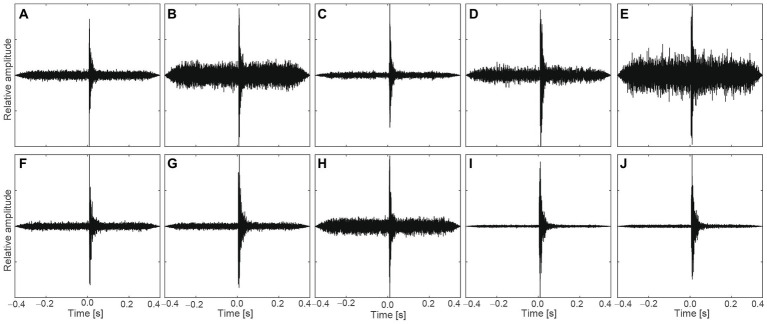
Sound **(A–J)**, temporal waveforms of impulsive sounds with background noises selected for the experiments.

Measurement of air-conditioner sounds generated by the inlet unit was carried out in an anechoic room. Domestic and split-type air conditioners were used for the measurement. The width, height, and depth of the air conditioner were 120, 34, and 26 cm, respectively. Squeaking sounds caused by the stick–slip phenomenon at the contact spots between the panels during defrosting were defined as impulsive sounds. This phenomenon was caused by the heat distortion of the exterior panels, which was related to the change in heat exchange temperature. The generated sound was recorded using a sound measurement system (SQuadrigaII, HEAD Acoustics, Aachen, Germany) and binaural microphone (BHS I, HEAD Acoustics). The sampling rate and resolution of the system were 48 kHz and 32 bits, respectively. The microphone was placed at a height of 1.6 m and a distance of 1.0 m from the air conditioner as shown in Figure 1. Ten impulsive sounds were extracted from the air conditioner sounds, as shown in Figure 2. To eliminate binaural effects, the recorded signals at the left and eight ears were transformed into monaural signals by mixing two signals with equal amplitudes.

Factors determined using the ACF have been proposed to evaluate various types of sound quality (Ando, 2009; Soeta and Ando, 2015). To determine the ACF factors, the normalized correlation functions of the transformed signals, *p*(t), as a function of the running step, *s*, were defined as:


(1) $$\phi \left(\tau \right)=\phi \left(\tau ;\;\;s,\;T\right)=\left(\frac{\Phi \left(\tau ;\;\;s,\;T\right)}{\sqrt{\Phi \left(\tau ;\;\;s,\;T\right)\Phi \left(\tau ;\;\;s,\;T\right)}}\right),$$

where:


(2) $$\Phi \left(\tau ;\;s,\;T\right)=\frac{1}{2T}\overset{s+T}{\underset{s-T}{\int }}p^{\prime }\left(t\right)p^{\prime }\left(t+\tau \right)dt.$$

When a monaural signal is used, Eq. (1) defines the normalized ACF. *2 T* is the integration interval, and *p*’(t) = *p*(t)*s_e_(t). *s*_e_(t) represents the impulse response of an A-weighted filter, which approximates ear sensitivity (Ando, 2009; Soeta and Ando, 2015). The normalized ACF was calculated using the geometric mean of the energy at *s* and the energy at *s* + *τ*.

L_Aeq_ was determined from the A-weighted *p*(t) as a function of the running step, *s*. L_Aeq_ was calculated using


(3) $$L_{Aeq}\left(s,T\right)=10log\sqrt{\Phi (0;\;\;s,\;\;T)}\;$$

**Figure 3:**
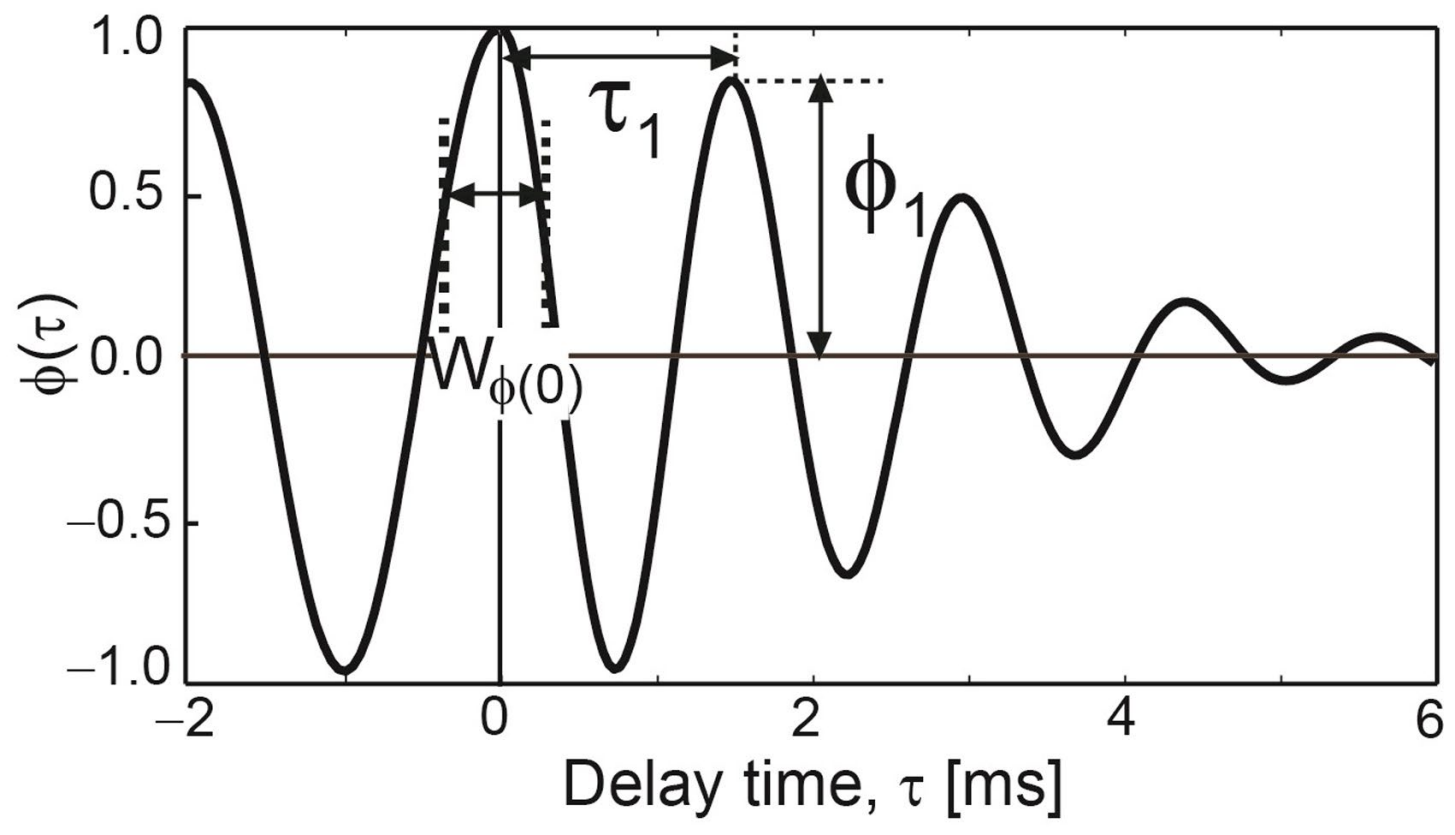
Definitions of autocorrelation function (ACF) factors, τ_1_, ϕ_1_, and W_ϕ(0)_.

**Figure 4:**
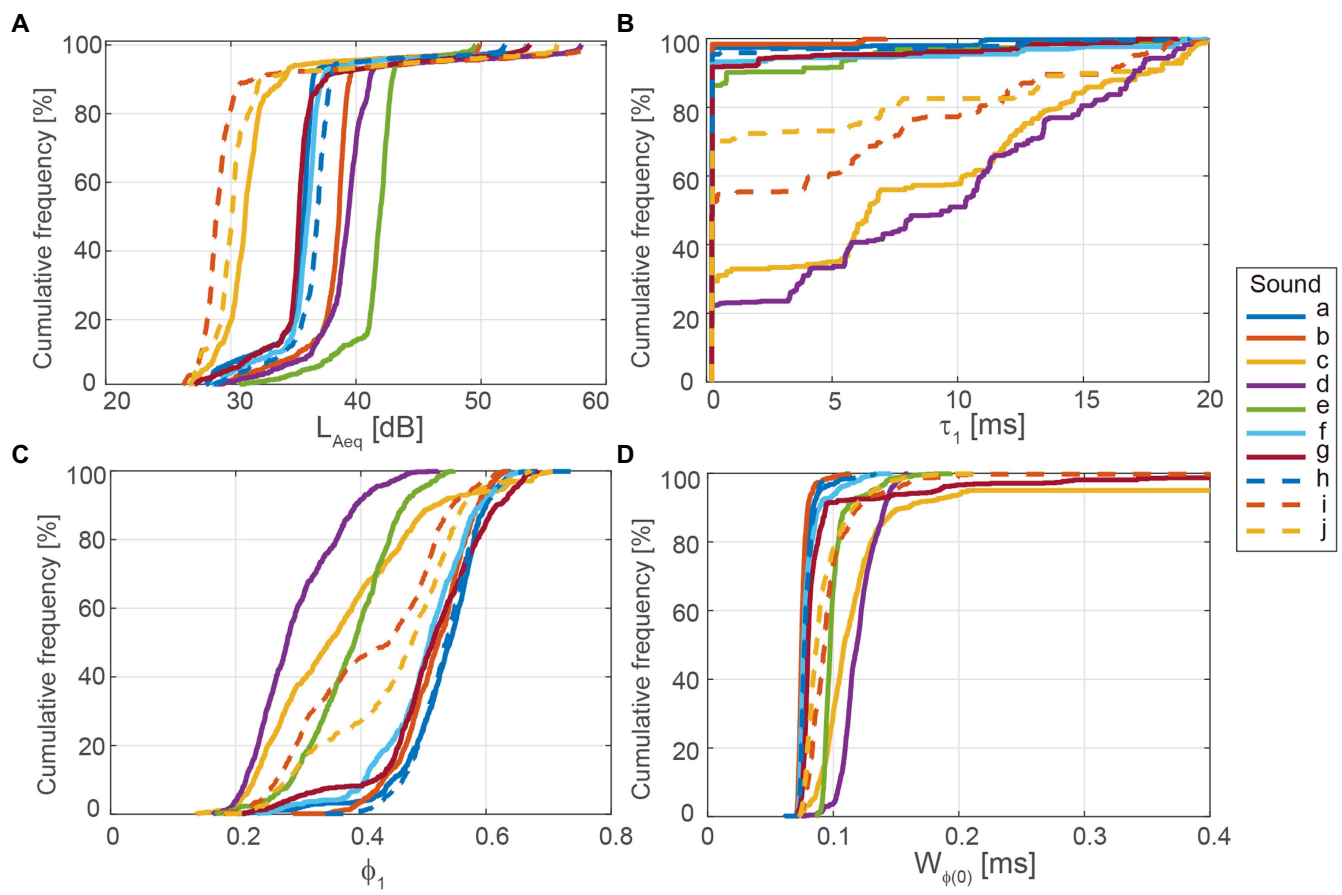
Cumulative frequency of autocorrelation function (ACF) factors for transient sounds used in the experiments. **(A)** L_Aeq_, **(B)** τ_1_, **(C)** ϕ_1_, and **(D)** W_ϕ(0)_.

This implies that L_Aeq_ is an ACF factor. The other ACF factors were determined from the normalized ACF, as shown in Figure 3. The delay time to the first maximum peak, τ_1_, corresponds to the fundamental frequency, that is, the perceived pitch. The amplitude of the first maximum peak, ϕ_1_, corresponds to the perceived pitch strength (Ando, 2009; Soeta and Ando, 2015). Higher τ_1_ and ϕ_1_ values mean that the sound has a lower and stronger pitch, respectively. The width of the first decay, W_ϕ(0)_, was defined as the delay time interval at a normalized ACF value of 0.5. W_ϕ(0)_ is the counterpart of the spectral centroid (Ando, 2009). Higher W_ϕ(0)_ values indicate that the sound includes more lower-frequency components. To evaluate the sound characteristics both quantitatively and qualitatively, L_Aeq_, τ_1_, ϕ_1_, and W_ϕ(0)_ were determined as functions of time. The integration interval, *2 T*, was 20 ms, and the running step, *s*, was 1 ms for all calculations. The analysis was performed using a MatLab-based program (Mathworks, Natick, MA, United States). The cumulative frequencies of all factors are shown in Figure 4.

### 2.2. Physiological experiments

The experimental procedures were approved by the ethics committee of the National Institute of Advanced Industrial Science and Technology (AIST) of Japan. All experimental participants gave written informed consent prior to participating in the experiment.

**Figure 5:**
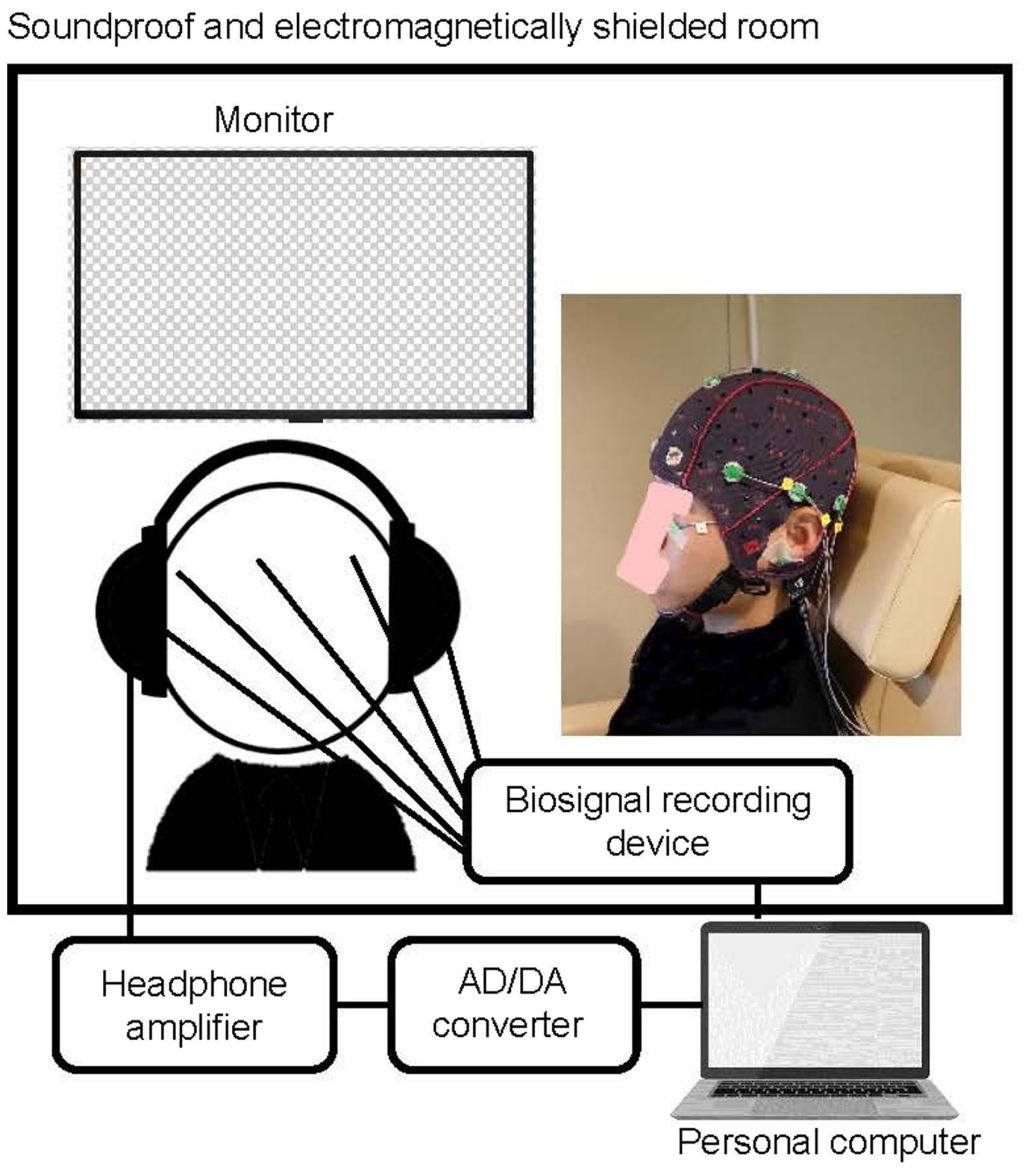
Experimental configuration of the physiological experiment.

A total of 14 individuals (eight men and six women) participated in the experiments. They were between 20 and 36 years old (median age, 21.0 years), with normal hearing and no history of neurological diseases. The experiment was carried out in a soundproof and electromagnetically shielded room. The physiological experimental setups are shown in Figure 5.

A total of 10 impulsive sounds, extracted from the measured air conditioner sounds in the anechoic room, were binaurally presented to the participants *via* a headphone (HD800, Sennheiser, Wedemark, Germany) and headphone amplifier (HDVD800, Sennheiser). The duration of each presented sound was 0.7 s, including 350 ms before and after the peak, as shown in Figure 2. All sounds were presented at the same L_Aeq_ ± of 1 dB as the actual measured L_Aeq_. L_Aeq_ was confirmed using a dummy head microphone (KU100, Neumann, Berlin, Germany) and sound calibrator (Type 4,231, Brüel & Kjær, Naerum, Denmark). The range of the L_Aeq_ was between 29.5 and 41.2 dB. The background noise level at the ear position in the room was 26.5 dBA. In the experiment, 10 impulsive sounds were presented in a randomized order with an inter stimulus interval randomly set between 1.0 and 1.5 s. To maintain an attentional level, participants were instructed to ignore the sound and to concentrate on a self-selected silent movie that was projected onto a monitor in front of them during the experiment.

**Figure 6:**
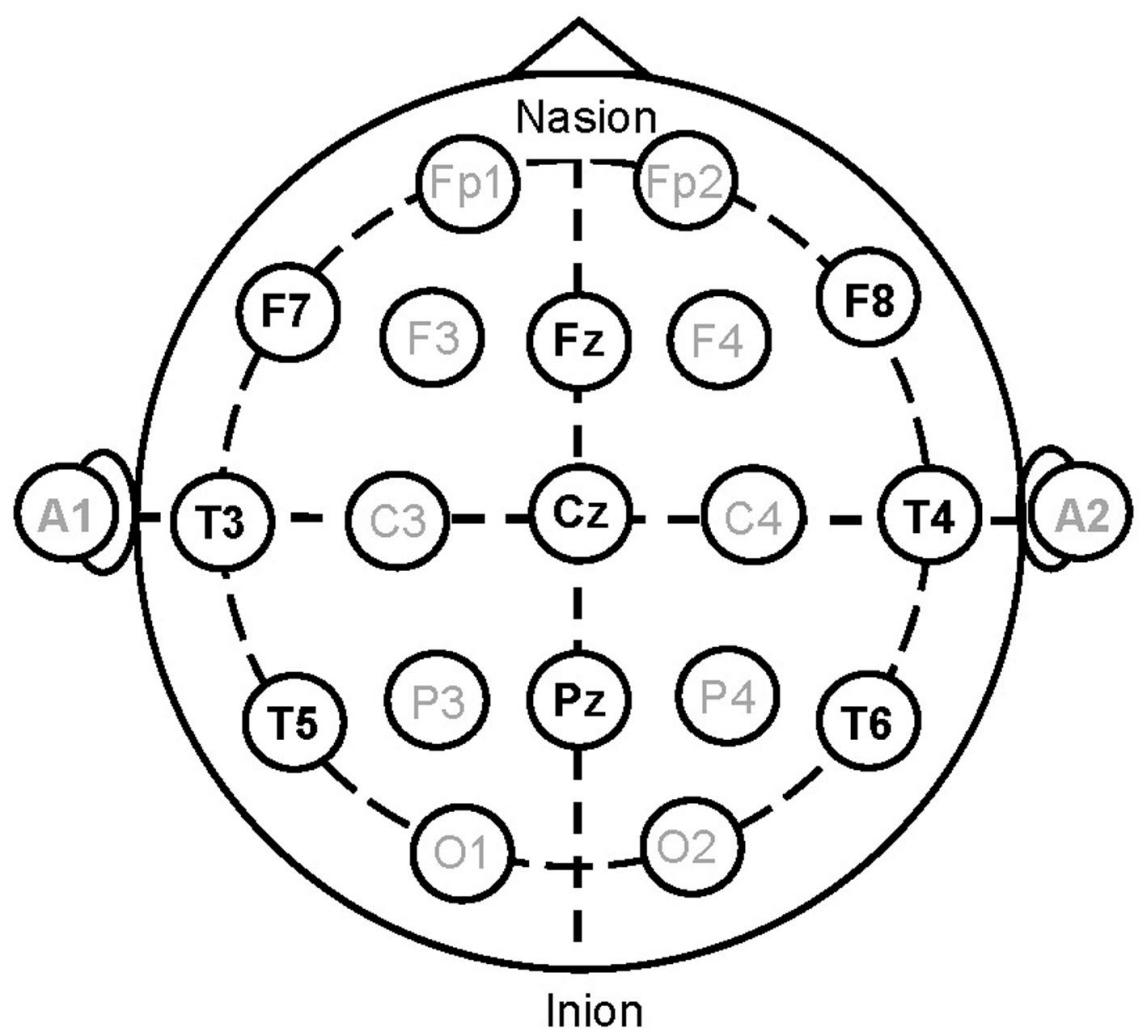
Electrode positions where EEG was measured.

Nine electroencephalography (EEG) electrodes were placed on the participants’ heads based on the International 10–20 System (F7, F8, T3, T4, T5, T6, Fz, Cz, and Pz) as shown in Figure 6. All EEG channels were referenced to linked ear lobes (A1 and A2), and the impedance was maintained below 20 kΩ. A ground electrode was placed on the forehead of each participant. EEG signals were amplified and recorded using a biosignal recording device (Polymate Pro MP6100; Miyuki Giken, Tokyo, Japan) with active electrodes. EEG data were digitized at a sample rate of 1,000 Hz and stored on the hard disc of a laptop computer.

The EEG data were averaged approximately 50 times. The average responses were digitally filtered between 1.0 and 30.0 Hz. The analysis time included the interval from 0.35 s prior to the peak of the impulsive sounds to 0.35 s after the peak of the impulsive sounds. The pre-stimulus period (average of 0.2 s prior to sound peak) was used as the baseline level. We obtained the N1 and P2 amplitudes for each electrode. First, the N1 and P2 responses, which were observed approximately 100 and 200 ms after the sound peak, were checked. Since clear N1 and P2 responses were not obtained from some participants and electrode positions, the root mean square (RMS) amplitudes between 0 and 300 ms were determined.

Correlation coefficients between the RMS amplitudes and ACF factors were calculated to determine the effects of ACF characteristics on brain response. The acoustic influence of impulsive sounds (10^th^-percentile values) and background noise (90^th^-percentile values) can be distinguished with the help of the ACF factors at different statistical levels. The effects of impulsive sounds on the RMS of amplitudes were statistically analyzed using repeated-measures analysis of variance (ANOVA).

## 3. Results

**Figure 7:**
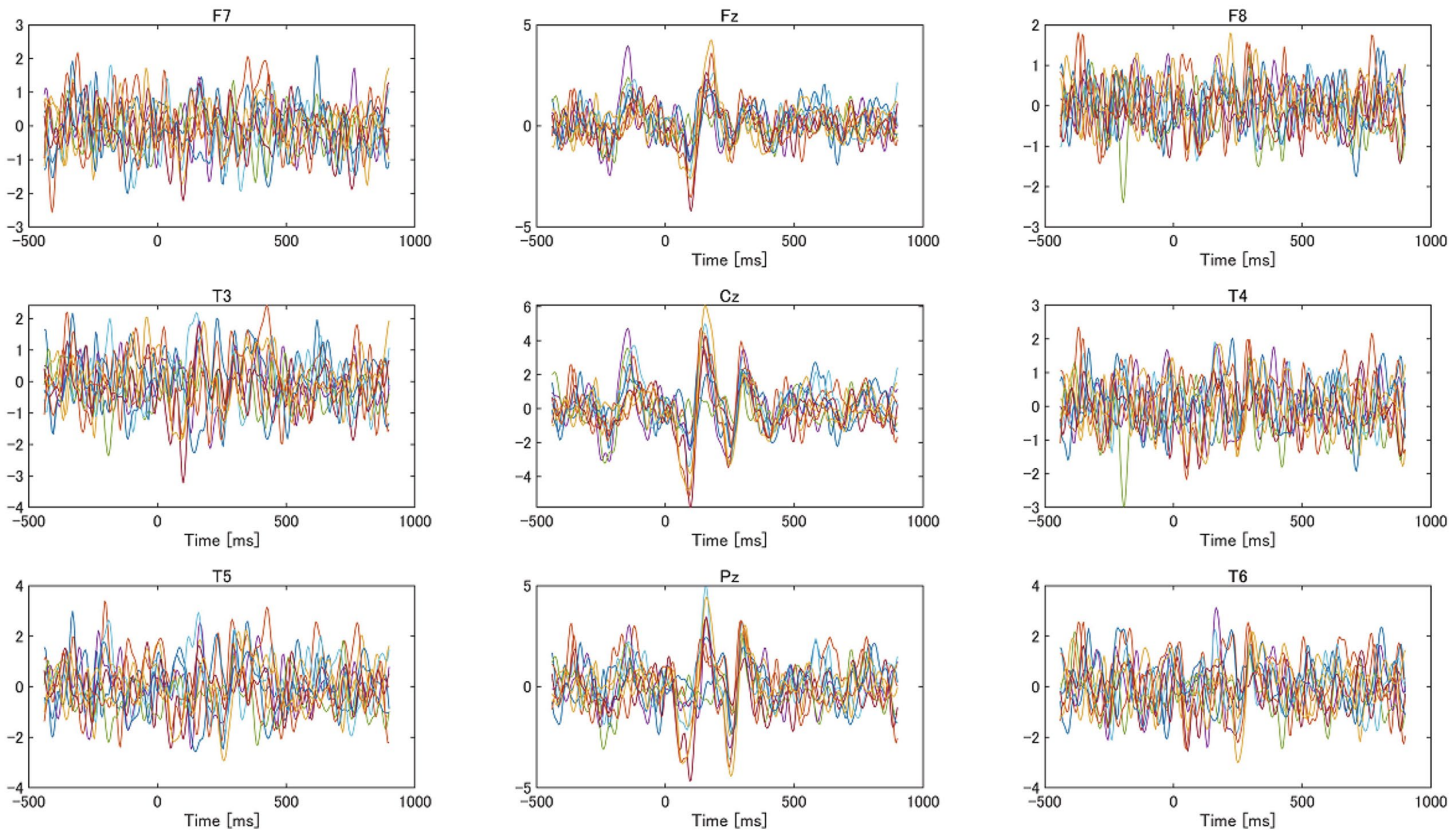
Auditory evoked responses (AEPs) observed at nine electrodes from a participant. The color of the lines indicates each AEP evoked by each impulsive sound.

Figure 7 shows the AEPs in response to the impulsive sounds observed at nine electrode positions for a participant. Notable N1 and P2 responses were observed at Fz, Cz, and Pz. Since many participants showed clear N1 and P2 responses at Fz and Cz, and as previous studies showed clear N1 responses at FCz (Dimitrijevic et al., 2008, 2009), located between Fz and Cz, we focused on the RMS response at Fz and Cz. In addition, we focused on the RMS responses at T5 and T6, which are approximately over the auditory cortex.

**Figure 8:**
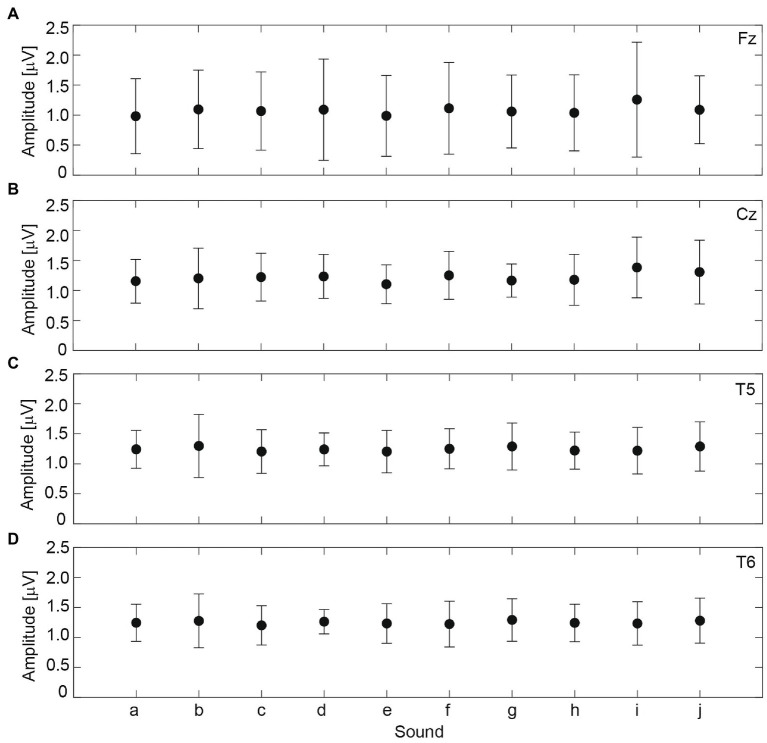
The mean root mean square (RMS) amplitude at electrode positions **(A)** Fz, **(B)** Cz, **(C)** T5, and **(D)** T6 from 14 participants. Each symbol indicates the averaged value, and error bars indicate standard deviations.

Figure 8 shows the mean RMS amplitudes at Fz, Cz, T5, and T6 for the 14 participants. Impulsive sound *i* yielded a maximum response at Fz and Cz, impulsive sound *b* yielded a maximum response at T5, and impulsive sound g yielded a maximum response at T6. Impulsive sound *e* exhibited a minimum response at Fz and Cz. The differences in the responses to each sound were small. ANOVA revealed that the effect of impulsive sound on the RMS amplitudes was not statistically significant at all electrode positions.

The correlation coefficients between the RMS amplitudes and ACF factors were determined, as listed in Table 1. The values of L_Aeq_10_ were significantly correlated with the RMS amplitudes at Cz (*p* < 0.05), T5 (*p* < 0.01), and T6 (*p* < 0.01). The values of τ_1_10_ were significantly and negatively correlated with RMS amplitudes at Fz (*p* < 0.05), Cz (*p* < 0.01), T5 (*p* < 0.01), and T6 (*p* < 0.01). The values of ϕ_1_10_ were significantly correlated with the RMS amplitudes at T5 (*p* < 0.05) and T6 (*p* < 0.05). The values of W_ϕ(0)_10_ were not significantly correlated with any RMS amplitudes.

**Table 1:** The correlation coefficients between ACF factors and the RMS amplitude at Fz, Cz, T5, and T6.

	L_Aeq_10_	τ_1_10_	ϕ_1_10_	W_ϕ(0) _10_	L_Aeq_10_ – L_Aeq_90_	τ_1_10_ – τ_1_90_	ϕ_1_10_ – ϕ_1_90_	W_ϕ(0) _10_ – W_ϕ(0) _90_
Fz	0.16	−0.18*	0.07	−0.07	−0.09	−0.03	0.06	−0.10
Cz	0.20*	−0.28**	0.09	−0.10	−0.02	−0.04	0.19*	−0.00
T5	0.26**	−0.28**	0.19*	−0.09	−0.07	−0.17*	0.06	−0.08
T6	0.30**	−0.28**	0.22*	−0.12	−0.07	−0.21*	0.08	−0.09

Asterisks show the statistical significance level, **p < 0.01, *p < 0.05.

## 4. Discussion

In the evaluation of the physiological responses to the impulsive sounds made by air conditioners, we found that the 10^th^-percentile values of the delay time and amplitude of the maximum peak of the ACF, τ_1_10_ and ϕ_1_10_, which correspond to the perceived pitch and pitch strength, correlated significantly with the average amplitudes of the AEPs.

In a previous study, τ_1_10_ values were significant predictors of subjective loudness and pitch for low-level impulsive sounds (Soeta et al., 2023). The τ_1_ and ϕ_1_ values were significant predictors of subjective annoyance to time-invariant air conditioner sounds (Soeta and Shimokura, 2017). Thus, our results suggested that τ_1_ and ϕ_1_ values can be significant factors for sound quality evaluations not only of time-invariant, but also of transient sounds.

The 10^th^-percentile values of L_Aeq_ were statistically correlated with the average amplitudes of the AEPs, suggesting that a larger peak of impulsive sounds caused larger brain responses. The amount of change from background sounds to the peak of impulsive sounds for L_Aeq_, L_Aeq_10_–L_Aeq_90_, was a significant predictor of subjective loudness (Soeta et al., 2023). Thus, it was assumed that the amount of change would also be a factor that correlated with brain responses. However, it was rejected. This might suggest that sound intensity itself is more dominant in the early phase of the cortex (Soeta and Nakagawa, 2009; Soeta and Nakagawa, 2010), where the basic characteristics of sounds are processed, and the amount of change in sound intensity is more dominant in the later phase of the cortex, where higher-order processing is fulfilled (Martin and Boothroyd, 2000; Dimitrijevic et al., 2009; Nishihara et al., 2011; He et al., 2012; Soeta and Nakagawa, 2012).

The amount of change from background sounds to the peak of the impulsive sounds for ϕ_1_ and ϕ_1_10_–ϕ_1_90_ was a significant predictor of subjective pitch and annoyance (Soeta et al., 2023). It was assumed that the amount of change was also a factor that correlated with brain response; however, it was rejected. This might suggest that pitch strength itself is also more dominant in the early phase of the cortex (Soeta et al., 2005, 2006; Soeta and Nakagawa, 2008) and the amount of change in pitch strength is more dominant in the later phase of the cortex, similar to L_Aeq_.

A limitation of this study is that the sound source was an air conditioner under different operational conditions, which provided only a narrow range of acoustic characteristics for impulsive sounds, and the number of participants. In future, the factors that characterize impulsive sounds and physiological responses, using a wider variety of sound sources and more participants, should be investigated to determine the allowable level for each factor under more realistic and reliable conditions. This will be beneficial to propose plausible standards for comfortable acoustic environments.

## 5. Conclusion

In this study, the AEPs in response to low-level impulsive sounds were analyzed to identify the physical factors that were significantly correlated with the responses. The results showed that the 10^th^-percentile values of L_Aeq_, the delay time, and the amplitude of the first maximum peak, τ_1_ and ϕ_1_, which correspond to the pitch and pitch strength, respectively, were significantly correlated with the amplitudes of the AEPs for low-level impulsive sounds. However, the amount of change from the background sound to the peak of the impulsive sounds was not significantly correlated with the amplitudes of the AEPs. This suggests that sound intensity, pitch, and pitch strength are more important than changes in the early phases of the cortex when people do not pay active attention to sounds. These results may provide a basis for further studies that can help define standards for achieving comfortable acoustic environments.

## Data availability statement

The raw data supporting the conclusions of this article will be made available by the authors, without undue reservation.

## Ethics statement

The studies involving human participants were reviewed and approved by the Ethics Committee of the National Institute of Advanced Industrial Science and Technology. The patients/participants provided their written informed consent to participate in this study.

## Author contributions

YS: conceptualization, methodology, software, writing—original draft, writing—review, and editing. EO: conceptualization, investigation, and data curation. All authors contributed to the article and approved the submitted version.

## Funding

This work was supported by Grant-in-Aid for Scientific Research (B) (grant nos. 18H03324 and 22H03916) from the Japan Society for the Promotion of Science.

## Conflict of interest

EO was employed by Mitsubishi Heavy Industries Ltd., Japan.

The remaining author declares that the research was conducted in the absence of any commercial or financial relationships that could be construed as potential conflicts of interest.

## Publisher’s note

All claims expressed in this article are solely those of the authors and do not necessarily represent those of their affiliated organizations, or those of the publisher, the editors and the reviewers. Any product that may be evaluated in this article, or claim that may be made by its manufacturer, is not guaranteed or endorsed by the publisher.
